# Quality of oxytocin ampoules available in health care facilities in the Democratic Republic of Congo: an exploratory study in five provinces

**DOI:** 10.7189/jogh.08.020415

**Published:** 2018-12

**Authors:** Peter Lambert, Tri-Hung Nguyen, Claire McEvoy, Rajpreet Singh Minhas, Philip Wright, Kim Deadman, Luc Mulimbalimba Masururu, Michelle P McIntosh

**Affiliations:** 1 Drug Delivery, Disposition and Dynamics, Monash Institute of Pharmaceutical Sciences, Monash University, Parkville, Victoria, Australia; 2 Mission in Health Care and Development, Luvungi MHCD Hospital, Uvira, Democratic Republic of Congo

## Abstract

**Background:**

Oxytocin injection is the first line therapy for the prevention and treatment of postpartum haemorrhage (PPH), the leading cause of maternal mortality. Currently access to high quality oxytocin in low and middle-income countries (LMICs) is compromised by variable manufacturing quality and the requirement for cold chain supply and storage to prevent product deterioration. Previous studies of oxytocin ampoules sampled from Africa, the region with highest maternal mortality rates, indicate that over half do not contain the specified amount of oxytocin. International efforts continue to further understand the issues relating to oxytocin quality in LMICs and this study is the first to assess oxytocin quality in the Democratic Republic of Congo (DRC), a country that bears one of the highest global rates of maternal mortality (693 maternal deaths per 100 000 live births). Importantly, the study methodology includes the use of investigative analytical techniques to understand the cause of quality deficiencies and inform remedial measures.

**Methods:**

The study involved sampling of oxytocin injection ampoules from public and private health care facilities (n = 15) in urban and rural areas within five provinces of the DRC. Where available, each sample comprised 20 ampoules of oxytocin injection (10 IU/mL) with smaller numbers collected where supplies were limited. Sample collection used overt sampling and mystery shopper approaches, as appropriate. Analysis of ampoules for oxytocin content and known degradation products utilised validated HPLC and LCMS methods, respectively. Sterility testing was conducted in accordance with the United States Pharmacopeia monograph.

**Results:**

Eighty percent of ampoules collected contained less than 90% of the specified content. Known degradation products of oxytocin were identified, indicating likely exposure to elevated temperatures post-manufacture. All samples contained an unknown impurity at a level of approximately 12.3% (8.0-20.5%) of the oxytocin main band peak. No samples failed sterility testing.

**Conclusions:**

There is evidence of a high prevalence of poor quality oxytocin ampoules in health facilities in the DRC likely resulting from both manufacturing quality issues and uncontrolled storage. A more comprehensive post-marketing surveillance study of oxytocin quality is warranted.

Postpartum haemorrhage (PPH) is the leading cause of maternal mortality, with these deaths occurring overwhelmingly in low and middle-income countries [1].

Injectable oxytocin, given intravenously (IV) or intramuscularly (IM), is recommended by the World Health Organisation (WHO) as the first line therapy for PPH prevention and treatment [2]. The temperature sensitivity of oxytocin in solution requires that the injectable product be supplied and stored under refrigerated (2-8°C) or cool (<25°C) conditions to minimise oxytocin degradation and maintain quality. However, in many low-resource countries ambient temperatures will exceed 25°C and cold chain infrastructure can be lacking or unreliable. Hogerzeil et al. showed the maximum temperature to which products were exposed during the shipment of essential commodities from Copenhagen to Lagos, Kampala and Bangkok was 33.6°C, 42.4°C and 37.5°C respectively [3]. More recently, the WHO conducted an observational study recording temperature variations along the oxytocin injection supply chain from manufacturer to points of use in Ghana and detected temperatures up to 30.1°C [4].

These supply chain challenges can have a detrimental impact on the quality of oxytocin injection available to the end user. A recent systematic review assessing the quality of oxytocin sampled from public and private supply chains in low and middle-income countries (LMICs) found the proportion of ampoules failing international specifications to be 57.5% and 22.3% in Africa and Asia respectively [5].

While exposure to elevated temperatures explains some quality issues with oxytocin injection products, deficiencies at the time of manufacture have also been identified. A post-marketing surveillance study in Ghana found that, in addition to 55.62% of ampoules failing content specifications, 87.5% of ampoules tested were shown to be non-sterile and two ampoules were identified as counterfeit products (ampoules containing no oxytocin) [6]. If maternal morbidity and mortality is to be better prevented, it is essential that the source and causes of substandard products be urgently identified. This is particularly the case in sub-Saharan Africa where the burden of maternal mortality and the prevalence of poor quality oxytocin are highest. In this region, constrained health care budgets can drive procurement of products based primarily on price, not quality, often from countries where regulatory oversight of manufacturers is variable. Additionally, local regulatory agencies do not have resources to conduct adequate quality control checks on these imported materials [7]. In this context, post-marketing surveillance studies can be a useful tool to identify quality issues towards which limited resources can be directed.

In collaboration with the local Ministry of Health (MoH) and the Mission for Healthcare and Development (MHCD), the objective of the study was to evaluate the quality of oxytocin ampoules (10 IU/mL) collected from a range of health care facilities across the Democratic Republic of Congo (DRC). The study is the first investigating oxytocin ampoule quality in the DRC and supports ongoing initiatives by the DRC MoH to address high rates of PPH in the country. The application of stability indicating methodology to identify and quantify known degradation products supports the differentiation of low content samples that have deteriorated post-manufacture from those resulting from poor manufacturing practices.

## METHODS

Oxytocin ampoules were sampled over a 14-day period in July 2016 from public and private health care facilities randomly selected in 15 locations (**Table 1**). Sampling was conducted by two representatives from the MHCD in accordance with the general principles of the WHO guidelines for survey of medicines quality [8]. Overt sampling was conducted at the MHCD hospitals in Kinshasa and Luvungi, with a mystery shopper approach used at all other locations. Following sampling, ampoules were stored in cool boxes to maintain refrigerated conditions. On return to Kinshasa the samples were refrigerated until shipment for analysis. A sample quantity of 20 ampoules per facility was targeted but not always available. Samples were shipped to Monash University, Melbourne, Australia, under ambient conditions (<72hrs) and tested for oxytocin content, quantification of known oxytocin degradants and sterility.

**Table 1:** Summary of sample collection sites and quantities

Sample No.	Province	Facility	Location	No. of ampoules per sample
1	Bandundu	Pharmacy	Rural centre	17
2	Bandundu	Health centre	Rural centre	20
3	Bandundu	Hospital	Rural centre	20
4	Bandundu	Pharmacy	Rural centre	20
5	Kongo Central	Health centre	Rural centre	19
6	Kongo Central	Pharmacy	Rural centre	20
7	Kongo Central	Clinic	Rural centre	10
8	South Kivu	Pharmacy	Urban	6
9	South Kivu	Pharmacy	Urban	20
10	South Kivu	Pharmacy	Urban	20
11	North Kivu	Pharmacy	Urban	20
12	South Kivu	Hospital	Rural centre	10
13	Kinshasa	Pharmacy	Urban	15
14	Kinshasa	Pharmacy	Urban	20
15	Kinshasa	Hospital	Peri-Urban	19

A high performance liquid chromatography method with UV detection (Shimadzu Nexera X2 UHPLC) was developed in order to quantify the amount of oxytocin present in each ampoule. This method was validated and used calibration standards to ensure appropriate within-run accuracy and precision. Five ampoules from each sample were tested.

Five ampoules from each sample were also quantitatively analysed for known oxytocin degradation products using a validated liquid chromatography – triple quadrupole mass spectrometry (LCMS) (Shimadzu LCMS-8030) assay.

Sterility testing was performed in accordance with US Pharmacopeia (USP) monograph (USP39) in an accredited laboratory in Melbourne, Australia (Chemical Analysis Pty Ltd) [9].

The study was approved by the Minister of Health, Democratic Republic of Congo and all results shared with local authorities and the Mission for Healthcare and Development. The study design was presented to the local Human Research and Ethics Committee (HREC) at Monash University, which deemed the study to be low risk and not requiring full review.

## RESULTS

As shown in **Table 1**, 15 facilities in five provinces of the DRC were sampled according to WHO-recommended methodology, including both urban and rural, public and private centres. While 15 facilities provided samples to the study, a large number of facilities approached (n>15) did not have oxytocin in stock and were not included in the study, however the details of these facilities were not recorded.

Between six and 20 ampoules were collected at each facility and ampoule markings specified the content of all samples (n = 256) to be 10 international units (IU) /mL (16.7 µg/mL). Samples collected came from four Chinese manufacturers, while two samples from pharmacies in Bukavu had no specified manufacturer and their provenance is unknown. Of the four known manufacturers, only one held a marketing authorisation to sell oxytocin in the DRC at the time of collection and no products collected were approved through WHO pre-qualification procedure.

None of the oxytocin ampoules collected were labelled for refrigerated storage, with labelling recommending ‘store in a cool place’ or similar and no samples were stored under refrigerated conditions at the time of collection. All ampoules collected were within the labelled expiry date, which ranged between Apr-17 to Nov-18 (**Table 2**).

**Table 2:** Summary of sample details and testing results

Sample No.	Province	Type of health facility	Date of manufacture	Expiry date	Mean ampoule content (%)	Sterility testing
1	Bandundu	Pharmacy	Nov-15	Nov-18	81.98	Passed
2	Bandundu	Health centre	Nov-15	Nov-18	82.78	Passed
3	Bandundu	Hospital	Nov-15	Nov-18	81.42	Passed
4	Bandundu	Pharmacy	Nov-15	Nov-18	81.78	Passed
5	Kongo Central	Health centre	Sep-15	Sep-18	103.62	Passed
6	Kongo Central	Pharmacy	Sep-15	Sep-18	104.10	Passed
7	Kongo Central	Clinic	Aug-15	Jul-18	79.95	Passed
8	South Kivu	Pharmacy	Sep-14	Sep-17	59.09*	Not Tested†
9	South Kivu	Pharmacy	Feb-15	Jan-18	77.67	Passed
10	South Kivu	Pharmacy	Nov-15	Oct-18	66.19	Passed
11	North Kivu	Pharmacy	Dec-14	Nov-17	79.66	Passed
12	South Kivu	Hospital	Feb-15	Jan-18	75.85	Passed
13	Kinshasa	Pharmacy	May-14	Apr-17	60.75	Passed
14	Kinshasa	Pharmacy	Nov-15	Nov-18	80.67	Passed
15	Kinshasa	Hospital	Nov-15	Nov-18	99.14	Passed

*Mean of three ampoules due to lack of available ampoules from this location.

†Not tested due to insufficient ampoules in sample.

Mean oxytocin content per ampoule as a percentage of the specified concentration of 16.7 µg/mL (n = 5 per sample) for each facility is presented in Table 2. Oxytocin content for individual ampoules ranged between 48% and 105% of the specified content. Eighty percent of all ampoules tested (ie, ampoules from 12 of 15 facilities) contained less than 90% of specified oxytocin content and were, therefore, outside international pharmacopoeial specifications. In the three facilities where mean ampoule oxytocin content was within specifications, all individual ampoules tested were also within specifications.

Samples ranged in age from 8 to 26 months post-manufacture at the time of collection, with no correlation observed between oxytocin content and the age of the ampoules collected. Ampoules from the same manufacturer and batch were found in multiple facilities, however, with varying oxytocin content. For example, all ampoules from a semi-urban, private hospital in Kinshasa met content requirements while those from the same batch sampled from a Kinshasa pharmacy and regional facilities in Bandundu province contained less than 90% of specified oxytocin content.

All samples tested passed sterility testing (see **Table 2**).

Further analysis indicated that all ampoules with less than the specified oxytocin content contained products known to be associated with the degradation of oxytocin [10]. The concentration of these degradants was inversely correlated with the content of oxytocin, suggesting issues with uncontrolled storage post-manufacture, rather than deficiencies at the time of manufacture, were the cause of low oxytocin content in these ampoules.

**Figure 1:**
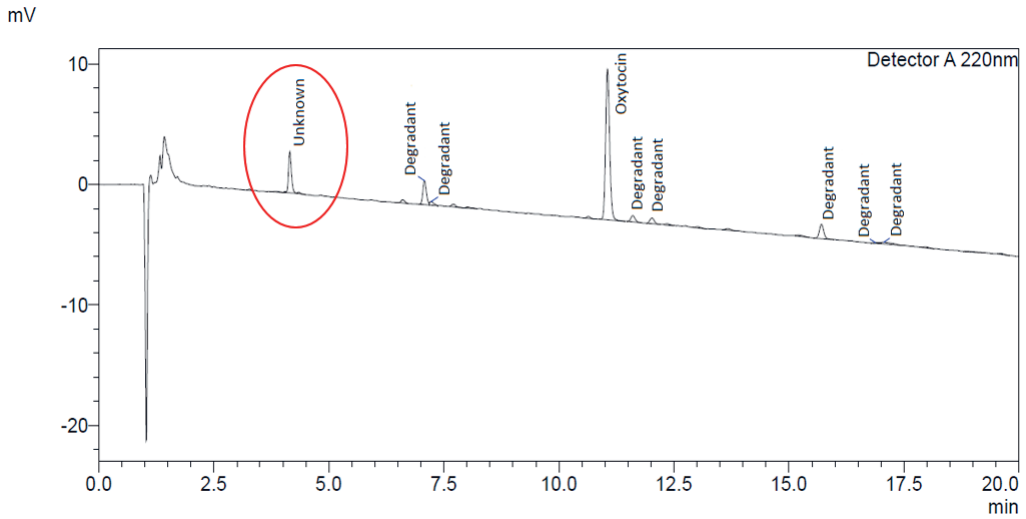
Example chromatogram - oxytocin content and degradant analysis.

In addition, the chromatography indicated the presence of an unknown compound at a mean level of 12.3% (8.0-20.5%) of the main oxytocin band in all ampoules from all manufacturers (**Figure 1**).

**Figure 2:**
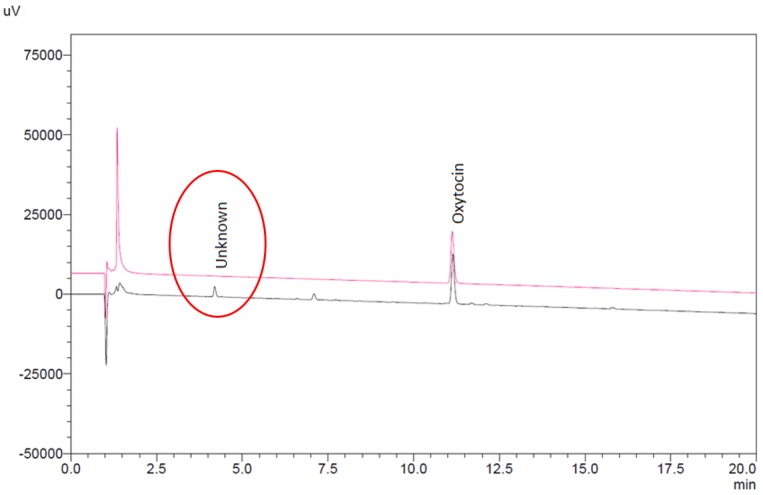
Comparison in chromatography of DRC-sourced oxytocin injection ampoules (lower chromatogram) with ampoules from a stringent regulatory authority approved, European-based supplier (upper chromatogram).

The impurity level did not correlate to oxytocin content or product age, while the chromatographic retention time of the compound did not correspond to any known degradation product of oxytocin. As a confirmatory measure, one sample of ampoules was also tested for oxytocin content in accordance with the product monographs in the United States and WHO International Pharmacopoeias. Both methods confirmed the presence of the unknown compound. By way of comparison, previous analysis of stringent regulatory authority approved oxytocin injection products from three European manufacturers showed no presence of this impurity (**Figure 2**).

## DISCUSSION

This study indicates that, in the regions of the DRC sampled, significant quality issues exist in relation to oxytocin supplies at the point of use, as a result of deficiencies in both product storage and at the point of manufacture. There is evidence of widespread product degradation, likely due to exposure to elevated temperatures along the supply chain. This is supported by results from a single batch of product sampled from multiple facilities in Kinshasa and Bandundu. The sample from a semi-urban hospital in Kinshasa was within specification while the oxytocin content of those ampoules of the same batch collected elsewhere were below specification, with the presence of degradation products of oxytocin indicating exposure to elevated temperatures. The number of samples showing evidence of deterioration was, perhaps, an unsurprising finding given that all products sampled were stored outside of the cold chain at the time of collection, and product labelling did not specify refrigerated storage.

That all products sampled were labelled for storage outside of the cold chain is problematic in the context of the DRC. While there are oxytocin injection products that have been shown to be stable for up to two years, if stored below 25°C (and have received marketing approvals supporting this storage condition), it has been shown that products stored outside of the cold chain in countries with tropical climates and constrained resources will likely be exposed to temperatures higher than 25°C [3,4]. The average maximum temperatures during July, when samples were collected, in the regions of the DRC included in this study, range from 25.°C in North Kivu to 30.3°C in Bandundu (www.climate-data.org). However, July is historically the coolest month of year and these regions can experience much higher temperatures at other times of the year. Consequently, the DRC is designated a zone IVa country (hot and humid climate) according to the WHO guidelines on stability testing of pharmaceuticals [11]. Under these guidelines it is a requirement that all pharmaceutical products supplied into zone IV countries, labelled for storage outside of the cold chain, demonstrate stability at 30°C for the duration of the specified product shelf-life. Based on current data, oxytocin injection ampoules stored at 30°C will degrade below 90% of specified content within 12 months, which does not represent a viable commercial shelf-life [12]. Therefore, all oxytocin injection product supplied into zone IV countries should be labelled for storage under cold chain conditions. To achieve change in this regard, local regulatory standards and procurement practices must support this requirement.

The presence of an unknown impurity in all samples, that appears unrelated to oxytocin content or product age, suggests an issue of manufacturing quality. To gain additional evidence towards this hypothesis, additional samples from one manufacturer were obtained from a central store in the DRC (ie, the product had not passed through the local supply chain). While all ampoules contained the specified content of oxytocin, the impurity was again present in all ampoules. Attempts to obtain samples direct from the manufacturer failed as the manufacturer was unwilling to supply this product to Australia.

An impurity content of the level detected exceeds international pharmacopoeial specifications and it is unlikely that any stringent regulatory authority would accept the presence of an impurity at this level in any drug product without comprehensive chemical characterisation and toxicological evaluation. Therefore, without these data, these results may indicate that the manufacturers identified do not meet international standards of Good Pharmaceutical Manufacturing Practice for this product. Given that all ampoules originated from China and contained the same impurity at similar levels, these products and/or their constituents may have similar provenance (despite being branded as different manufacturers). An investigation to identify this impurity and the potential implications is ongoing and will be detailed in a subsequent publication.

Of the products sampled, only one manufacturer held a marketing authorisation from the local regulatory authority to supply oxytocin into the DRC at the time of collection. While it is unclear whether any of the sampled products were procured legally under a waiver from the local regulators, this finding aligns with previous studies indicating that the procurement of unregistered products in both the public and private sectors of sub-Saharan countries is widespread [6,13].

Finally, it should be noted that many facilities approached held no stocks of oxytocin, indicating that in these regions, oxytocin availability is limited, presenting issues of access in addition to those of product quality.

### Strengths and limitations

The use of a stability-indicating analytical method that enabled quantification of the known degradants of oxytocin within the product was a study strength. This provided supportive evidence of degradation, allowed the detection of the unknown impurity and is recommended for future studies. A mystery shopper approach was employed at the majority of sample sites which reduced the likelihood of provider bias and ensured representative samples from the facilities visited.

In terms of limitations, the number of facilities from which samples were collected was small and these results should not be considered representative of the national situation. Also, it was not possible to ship the samples collected from the DRC to the testing facility under refrigerated conditions, presenting the possibility of oxytocin degradation during shipping. However, the duration of storage outside of the cold chain was limited and the samples were insulated to limit exposure to temperature extremes. The known temperature dependent degradation kinetics of oxytocin in solution would indicate that samples maintained under these conditions for such a limited period (<72 hours) would not undergo any significant deterioration during shipment [12]. This is supported by a number of these samples, when tested, meeting oxytocin content specifications without evidence of degradation.

## CONCLUSIONS

This study provides evidence of oxytocin injection quality issues in samples collected from a small number of public and private health care facilities in five provinces of the DRC. The results indicate that there is significant degradation of oxytocin, likely due to heat exposure, and procurement of low quality, often unregistered, products with inappropriate labelling for the region. As the first study conducted in the DRC, these results indicate that a more comprehensive post-marketing surveillance investigation of oxytocin injection quality is warranted to support local and international efforts to improve product quality and maternal health outcomes.
